# Experimentally Self-Testing Partially Entangled Two-Qubit States on an Optical Platform

**DOI:** 10.3390/e28010079

**Published:** 2026-01-09

**Authors:** Xin Zhao, Yan-Han Yang, Li-Ming Zhao, Ming-Xing Luo

**Affiliations:** School of Information Science and Technology, Southwest Jiaotong University, Chengdu 610031, China; 2023210925@my.swjtu.edu.cn (X.Z.); yangyanhan@swjtu.edu.cn (Y.-H.Y.)

**Keywords:** quantum entanglement, self-testing, photonic entanglements

## Abstract

We demonstrate a complete and experimentally validated self-testing protocol for two-qubit partially entangled states, which avoids the need for full tomographic reconstruction. Using a room-temperature type-II PPKTP polarization-entangled source and a free-space optical setup, we implement both quantum state tomography and optimal generalized Bell measurements within a single system. Our approach achieves high-fidelity self-testing of non-maximally entangled states under black-box assumptions, establishing a solid foundation for device-independent quantum information processing applications.

## 1. Introduction

Quantum entanglement serves as a fundamental resource in quantum information science, providing key advantages for quantum communication and computing that surpass classical limits. The presence and quality of entanglement directly determine the ultimate performance and security of quantum protocols [1,2]. However, conventional quantum state tomography faces significant limitations: it requires an exponentially growing number of measurements with system size and relies on perfectly calibrated, trusted measurement devices [3,4]. These device-dependent assumptions and scalability issues present major obstacles for reliable quantum state verification.

As quantum systems increase in complexity, entanglement verification encounters additional challenges. Under realistic conditions with noise and loss, entanglement fidelity decays exponentially with transmission distance or qubit number [5]. Many-body quantum systems exhibit complex entanglement structures that are difficult to characterize using bipartite or local frameworks [6]. Experimental platforms also face significant trade-offs between entanglement generation rates and scalability [7]. Addressing these challenges is essential for realizing practical quantum networks and advancing our understanding of quantum many-body systems [8,9,10].

Quantum state self-testing has emerged as a highly robust device-independent protocol that overcomes these limitations [11]. Based solely on quantum mechanical constraints, this approach uniquely certifies prepared states and measurement operators (up to local isometries) without requiring trusted or calibrated measurement devices [12]. The method leverages Bell nonlocality: by analyzing correlation data and Bell inequality violations, one can infer the system’s entanglement structure and identify specific target states [13,14]. As the highest security level in device-independent verification, self-testing provides resistance against side-channel attacks and establishes connections between fundamental nonlocality and practical security protocols [15,16,17].

Agresti et al. experimentally demonstrated device-independent self-testing of multi-source quantum network states for the first time, certifying both parallel and star network configurations [18]. Their work introduced self-testing protocols with a constant number of measurement settings independent of network size, provided the first device-independent lower bounds on network-state fidelity using the swap method and semidefinite programming, and employed both the Hoeffding and Azuma-CHoeffding inequalities to ensure reliable statistical analysis even in the presence of memory effects. In parallel, Xu et al. achieved the first experimental device-independent self-testing of multiphoton graph states, using scalable Bell inequalities with measurement complexity growing only linearly with the number of qubits, and demonstrated robustness by observing Bell violations above the self-testing threshold under realistic noise [19]. Supported by high-fidelity parametric down-conversion sources and independent stabilizer-based fidelity verification, these experiments show strong agreement with theoretical predictions and establish a solid experimental foundation for device-independent certification of complex multipartite quantum systems.

This work systematically investigates self-testing schemes based on Bell inequalities, designs generalized inequalities using the sum-of-squares method, and experimentally validates their effectiveness for certifying specific entangled states [20]. We evaluate the method’s applicability and accuracy across different quantum states, discuss its practical advantages and limitations, and assess its potential for enhancing security and efficiency in quantum information technologies.

## 2. Theoretical Framework

### 2.1. Quantum Nonlocality

Quantum nonlocality provides the theoretical foundation for quantum state self-testing. The foundational debate began with the 1935 EPR argument, which questioned the completeness of quantum mechanics under the principle of local realism [21]. Bell subsequently transformed this philosophical discussion into a physically testable framework by demonstrating that any local hidden variable theory must satisfy certain constraints now known as Bell inequalities [22]. Beginning with the pioneering experiments by Aspect et al. [23], numerous studies have consistently observed violations of these inequalities. These experimental results confirm that the correlations exhibited by quantum entanglement cannot be explained by any local hidden variable model, representing an intrinsically nonlocal phenomenon. This fundamental nonlocality enables the inference of essential properties of unknown quantum states through statistical analysis of correlation data alone, thereby establishing the theoretical basis for quantum state self-testing.

### 2.2. Quantum Self-Testing

Building upon the concept of quantum nonlocality, quantum state self-testing emerges as a powerful device-independent certification method. The core principle relies on the observation that certain experimentally measured correlations cannot be described by local hidden variable models. Such models assume that all correlations originate from some shared hidden variable λ distributed according to ρ(λ), such that the joint probability decomposes as

(1) $$\begin{array}{c} p\left(a,b|x,y\right)=\int d\lambda \rho \left(\lambda \right)p_{A}\left(a|x,\lambda \right)p_{B}\left(b|y,\lambda \right). \end{array}$$

When experimentally observed correlations significantly deviate from this structure and further satisfy a uniqueness condition—meaning that only a specific quantum state |ψ〉 and measurement operators $\left\{M_{a|x}\right\}$, $\left\{M_{b|y}\right\}$ (up to local isometries) can reproduce the observed distribution—we can conclusively assert that the system has realized the target quantum state and measurements.

**Figure 1:**
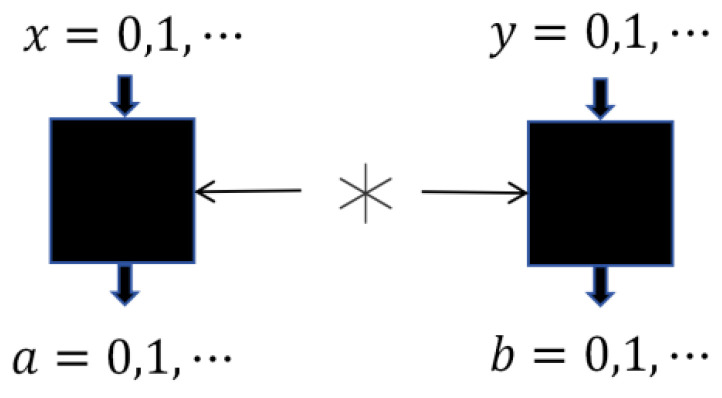
Schematic representation of the black-box approach for self-testing.

In the device-independent paradigm, the entire experimental setup is treated as an uncharacterized black box, as illustrated in Figure 1. Within this framework, Alice and Bob receive inputs *x* and *y*, respectively, and return outputs *a* and *b* based on their measurements. The joint probability distribution p(a,b|x,y) constitutes the only accessible data. If this distribution can be realized by some quantum state |ψ〉 and measurement operators such that

(2) $$\begin{array}{c} p\left(a,b|x,y\right)=\langle \psi |M_{a|x}⊗M_{b|y}|\psi \rangle , \end{array}$$

then the objective of self-testing is to demonstrate that this quantum realization is essentially unique up to local isometries.

For the specific case where the shared state is the Bell state $|\Phi^{+}\rangle$ and the measurements are optimally chosen for the CHSH inequality, the correlation strength reaches the Tsirelson bound of $2\sqrt{2}$. Table 1 provides the complete set of ideal conditional probabilities p(a,b|x,y) for this scenario. Any experimental realization that reproduces this distribution within acceptable error margins consequently enables device-independent certification of entanglement.

To implement this black-box approach experimentally, photonic polarization encoding provides an ideal physical platform [24,25,26]. This encoding scheme maps the computational basis states |0〉 and |1〉 to horizontal polarization |H〉 and vertical polarization |V〉, respectively, offering the dual advantages of low decoherence and capability for high-speed random setting switching. Furthermore, the required optical components—including wave plates and polarizing beam splitters—have reached a high degree of integration. In our experiments, we therefore adopt polarization encoding, which naturally maps Pauli operators to specific measurement configurations as $\sigma_{Z}=\left|H\rangle \langle H\right|-\left|V\rangle \langle V\right|$ and $\sigma_{X}=\left|+\rangle \langle +|-|-\rangle \langle -\right|$, where $|\pm \rangle =(|H\rangle \pm |V\rangle )/\sqrt{2}$ forms the complementary basis. Alice employs setting x=0 to implement $\sigma_{Z}$ and x=1 for $\sigma_{X}$, while Bob follows an analogous measurement strategy. The ±1 outcomes from these measurements generate the joint probability distributions necessary for demonstrating violation of Bell inequalities.

### 2.3. CHSH Inequality

Among various Bell inequalities, the CHSH inequality [27] holds particular importance for self-testing due to its conceptual simplicity and experimental feasibility. This inequality applies to bipartite scenarios where each party has two measurement settings and two possible outcomes, taking the form

(3) $$\begin{array}{c} S=A_{0}B_{0}+A_{0}B_{1}+A_{1}B_{0}-A_{1}B_{1}, \end{array}$$

where $A_{x}$ and $B_{y}$ represent observables with possible outcomes of ±1. While local hidden variable theories are constrained to yield values |S|≤2, quantum mechanics permits violations up to the Tsirelson bound of $2\sqrt{2}$. This maximum quantum value is achieved only under two specific conditions:

First, Alice and Bob must share the maximally entangled Bell state $|\Phi^{+}\rangle =(|HH\rangle +|VV\rangle )/\sqrt{2}$. Second, their measurement operators must be chosen as

(4) $$\begin{array}{c} {\hat{A}}_{0}=\sigma_{Z},\;{\hat{A}}_{1}=\sigma_{X},\\ {\hat{B}}_{0}=\left(\sigma_{Z}+\sigma_{X}\right)/\sqrt{2},\;{\hat{B}}_{1}=\left(\sigma_{Z}-\sigma_{X}\right)/\sqrt{2}. \end{array}$$

Here, ${\hat{A}}_{x}$ and ${\hat{B}}_{y}$ denote the x-th measurement operator of Alice and the y-th measurement operator of Bob, respectively. The correlation expectations $E_{xy}=\langle \Phi^{+}|{\hat{A}}_{x}⊗{\hat{B}}_{y}|\Phi^{+}\rangle$ for these measurement settings, as summarized in Table 2, yield the CHSH combination $S=|E_{00}+E_{01}+E_{10}-E_{11}|=2\sqrt{2}$. Consequently, any experimental observation of a CHSH value approaching this theoretical maximum provides strong evidence for the certification of $|\Phi^{+}\rangle$ within the device-independent framework.

The device-independent paradigm extends beyond the standard CHSH inequality to a broader class of quantum states via generalized Bell inequalities, requiring that the maximal quantum violation $S_{\mathrm{QM}}$ uniquely determines the underlying quantum realization. Consequently, when the experimentally observed value $S_{\exp}$ approaches $S_{\mathrm{QM}}$, the fidelity *F*—which quantifies the closeness between the unknown prepared state ρ and an ideal target pure state |ψ〉—satisfies F≥1−ϵ, where ϵ decreases linearly with the deviation $\left|S_{\mathrm{QM}}-S_{\exp}\right|$ [28,29,30,31,32,33]. Formally, with the target state’s density matrix σ=|ψ〉〈ψ|, the fidelity is defined as

(5) $$\begin{array}{c} F\left(\rho ,\sigma \right)={\left[\mathrm{Tr}\left(\sqrt{\sqrt{\rho }\;\sigma \;\sqrt{\rho }}\right)\right]}^{2}, \end{array}$$

ranging from 0 to 1, where higher values indicate that ρ is closer to |ψ〉.

To construct such Bell expressions for arbitrary target states, a variational approach has been developed [34]. Although the variational method provides necessary conditions for maximal violation, it may fail to identify global maxima since local extrema do not necessarily correspond to Tsirelson bounds. This limitation is effectively addressed by the more robust sum-of-squares method. Applying this methodology yields a generalized Bell operator:

(6) $$\begin{array}{c} S:=\frac{A_{1}B_{1}+A_{1}B_{2}}{2\cos b_{\theta }}+s_{2\theta }\frac{A_{2}B_{1}-A_{2}B_{2}}{2\sin b_{\theta }}+\frac{1}{2}c_{2\theta }\left(A_{1}+\frac{B_{1}+B_{2}}{2\cos b_{\theta }}\right) \end{array}$$

with a corresponding verification protocol:

(7) $$\begin{array}{c} \frac{\langle {\hat{A}}_{1}{\hat{B}}_{1}\rangle +\langle {\hat{A}}_{1}{\hat{B}}_{2}\rangle }{2\cos b_{\theta }}+s_{2\theta }\frac{\langle {\hat{A}}_{2}{\hat{B}}_{1}\rangle -\langle {\hat{A}}_{2}{\hat{B}}_{2}\rangle }{2\sin b_{\theta }}\\ +\frac{1}{2}c_{2\theta }\left(\langle {\hat{A}}_{1}\rangle +\frac{\langle {\hat{B}}_{1}\rangle +\langle {\hat{B}}_{2}\rangle }{2\cos b_{\theta }}\right)\leq 2, \end{array}$$

where $b_{\theta }=\frac{\pi }{2}-\arctan\left(\sqrt{\frac{1+c_{2\theta }^{2}/2}{s_{2\theta }^{2}}}\right)$, with $s_{2\theta }=\sin\left(2\theta \right)$ and $c_{2\theta }=\cos\left(2\theta \right)$. The optimal measurement settings are given by

(8) $$\begin{array}{c} {\hat{M}}_{1}^{\left(1\right)}=\sigma_{Z},\;{\hat{M}}_{2}^{\left(1\right)}=\sigma_{X},\\ {\hat{M}}_{y}^{\left(2\right)}=\cos\left(b_{\theta }\right)\sigma_{Z}-{\left(-1\right)}^{y}\sin\left(b_{\theta }\right)\sigma_{X}. \end{array}$$

The local bound for this inequality, achievable through the deterministic strategy $\left\{A_{1}=A_{2}=B_{1}=1,B_{2}=-1\right\}$, is

(9) $$\begin{array}{c} S=\frac{c_{2\theta }}{2}+\sqrt{\frac{27+8c_{4\theta }+c_{8\theta }}{8-8c_{4\theta }}}. \end{array}$$

where $c_{2\theta }=\cos\left(2\theta \right)$, $c_{4\theta }=\cos\left(4\theta \right)$, and $c_{8\theta }=\cos\left(8\theta \right)$.

The establishment of fidelity lower bounds from observed the value of S constitutes the core verification mechanism of self-testing [12]. In our experimental implementation, we have developed a polarization-encoded system capable of preparing the family of states |ψ(θ)〉=cosθ|HH〉+sinθ|VV〉. Through a measurement module incorporating precisely controlled wave plates and polarizing beam splitters, we execute the generalized Bell measurements required for self-testing, thereby enabling simultaneous determination of both state fidelity and the value of S with high precision.

## 3. Experimental Setup

This study demonstrates device-independent self-testing of high-fidelity quantum states through violations of generalized Bell inequalities. The experimental implementation comprised four sequential components:

1. Generation of near-ideal polarization-entangled photon pairs via type-II spontaneous parametric down-conversion (SPDC);
2. Precise configuration of optimal measurement bases for generalized Bell inequalities corresponding to different target states;
3. Establishment of statistical significance through coincidence counting with rigorous error control;
4. Estimation of fidelity lower bounds based on the observed value of S, providing quantitative validation of the self-testing protocol.

To systematically validate the effectiveness of the proposed self-testing protocol for partially entangled states, we designed an experimental sequence incorporating a built-in benchmark control. Three representative quantum states were prepared with parameters $\theta =30^{\circ }$, $45^{\circ }$, and $60^{\circ }$. Among them, the state with $\theta =45^{\circ }$ corresponds to a maximally entangled Bell state (e.g., $|\Phi^{+}\rangle$), whose entanglement properties are theoretically well characterized, including the ability to achieve the maximal Bell violation. This state is therefore designated as the benchmark control, serving to (i) verify that the experimental setup and self-testing protocol can attain the expected theoretical performance under ideal entanglement conditions, (ii) calibrate systematic effects such as overall efficiency, alignment accuracy, and noise in the experimental system, and (iii) provide a reference for comparative analysis of the two partially entangled test states with $\theta =30^{\circ }$ and $60^{\circ }$. Through this benchmark–Ctest design, we can clearly identify the performance limits of the self-testing protocol under ideal conditions and assess its robustness and applicability to more general, non-maximally entangled scenarios within a coherent experimental framework.

### 3.1. Entanglement Source Preparation

**Figure 2:**
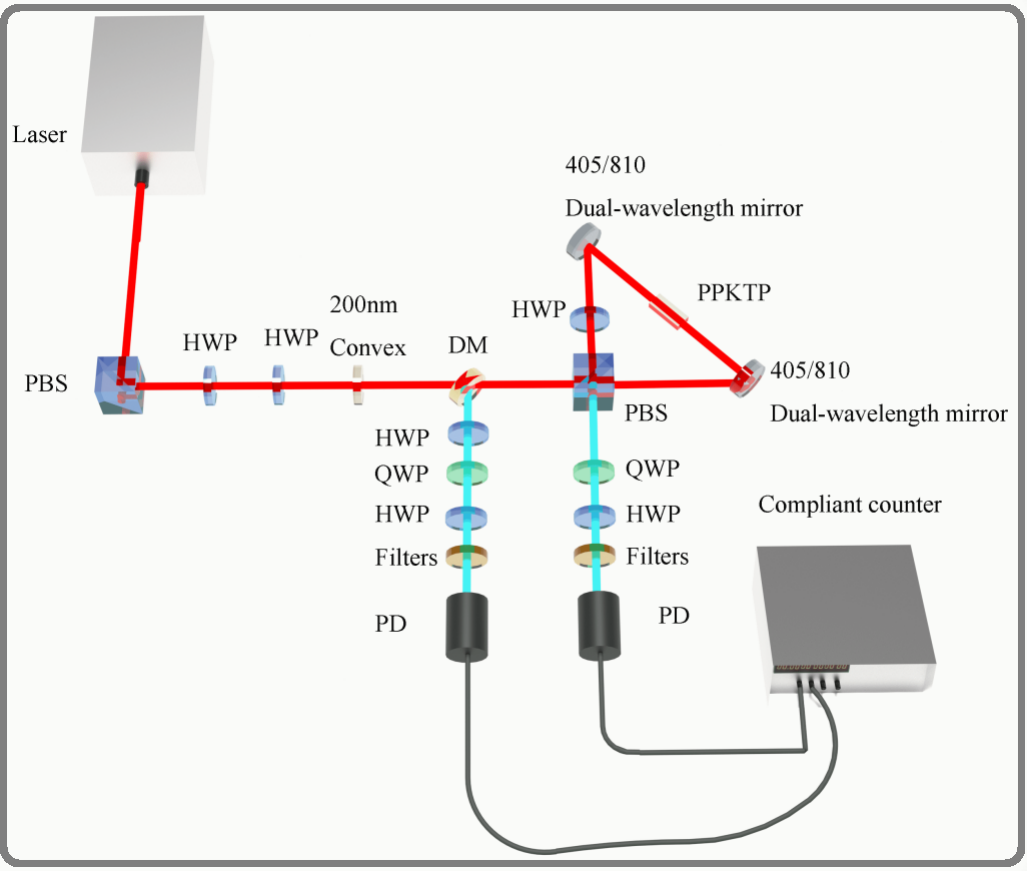
Schematic of the experimental setup. A 405 nm pump laser, with polarization controlled by two half-wave plates (HWPs), enters a Sagnac interferometer containing a PPKTP crystal. Orthogonally polarized photon pairs at 810 nm are generated via spontaneous parametric down-conversion (SPDC). A polarizing beam splitter (PBS) and dichroic mirror (DM) separate photon pairs into distinct paths, with quantum state measurement accomplished through quarter-wave plate (QWP) and HWP combinations. Key components: half-wave plates (HWPs), quarter-wave plates (QWPs), polarizing beam splitters (PBSs), dichroic mirrors (DMs), and spectral filters.

The experimental architecture, depicted in Figure 2, employs polarization-encoded photon pairs as qubits, where all optical components and their functions are shown in Table 3. The system integrates three functional modules—an entangled photon pair source, a state preparation unit, and a projective measurement unit—collectively enabling quantum state generation, manipulation [35,36,37,38,39], and measurement implementation.

The state preparation protocol begins with a vertically polarized laser beam $|\psi \rangle =|V\rangle ={\left[0,1\right]}^{T}$ generated by the source. After passing through a PBS, the beam undergoes sequential wave-plate transformations: a first HWP at angle θ yields $|\psi \left(\theta \right)\rangle ={\left[-\sin\theta ,-\cos\theta \right]}^{T}$, followed by a second HWP at $45^{\circ }$ producing $|\psi \left(\theta \right)\rangle ={\left[\cos\theta ,\sin\theta \right]}^{T}=\cos\theta |H\rangle +\sin\theta |V\rangle$.

The beam then traverses a DM and reaches a second PBS, where it splits into two paths. In the upper path, the |V〉 component passes through a third HWP ($45^{\circ }$), converting to |H〉, and then interacts with the PPKTP crystal via type-II SPDC to probabilistically generate signal ($|H_{s}\rangle$) and idler ($|V_{i}\rangle$) photons. The third PBS transmits $|H_{s}\rangle$, which reflects from a DM and propagates leftward, with a fourth HWP ($45^{\circ }$) converting it to $|V_{s}\rangle$. Simultaneously, $|V_{i}\rangle$ propagates vertically to the right path.

In the lower path, transmission through the third PBS precedes similar PPKTP interaction. The third HWP converts generated $|H_{s}\rangle$ to $|V_{s}\rangle$ and $|V_{i}\rangle$ to $|H_{i}\rangle$. These components recombine at the second PBS: $|V_{s}\rangle$ reflects vertically, passes through the DM to the left path, and converts to $|H_{s}\rangle$ via the fourth HWP; $|H_{i}\rangle$ transmits directly to the right path.

The resulting entangled state becomes

(10) $$\begin{array}{c} \left|\psi \left(\theta \right)\rangle =\cos\theta \right|H_{s}H_{i}\rangle +\sin\theta |V_{s}V_{i}\rangle \end{array}$$

This completes preparation of the target two-qubit entangled state |ψ(θ)〉=cosθ|00〉+sinθ|11〉. Output port wave plates enable fine phase and amplitude adjustments for high-fidelity state preparation. Subsequent projective measurements employ a measurement unit comprising QWPs, HWPs, and polarizing beam splitters.

The polarization transformation matrices are defined as

(11) $$\begin{array}{c} \mathrm{HWP}\left(h\right)=\left(\begin{array}{cc} \cos(2h) & -\sin(2h)\\ -\sin(2h) & -\cos(2h) \end{array}\right),\\ \mathrm{QWP}\left(q\right)=\left(\begin{array}{cc} 1-i\cos(2q) & \;isin(2q)\\ \;isin(2q) & 1+i\cos(2q) \end{array}\right) \end{array}$$

A coincidence counting system records four-fold coincidence events, enabling simultaneous quantum state tomography characterization and device-independent self-testing verification.

### 3.2. Measurement Setup

The measurement apparatus employs cascaded QWP, HWP, and PBS elements to implement arbitrary polarization rotations on the Bloch sphere. This configuration facilitates projective measurements in any desired basis and enables complete density matrix reconstruction. The compact, fully passive module exhibits high mechanical stability, with basis calibration errors $\leq 0.2^{\circ }$ during extended measurement periods, satisfying precision requirements for high-fidelity entanglement verification.

The QWP-HWP combination enables basis transformations from {|H〉,|V〉} to $\{|B_{+}\rangle ,|B_{-}\rangle \}$, where $|B_{+}\rangle =\cos(\frac{b_{\theta }}{2})|H\rangle +\sin\frac{b_{\theta }}{2}|V\rangle$ and $|B_{-}\rangle =\sin(\frac{b_{\theta }}{2})|H\rangle -\cos\frac{b_{\theta }}{2}|V\rangle$:

(12) $$\begin{array}{ccc} & & |B_{+}\rangle =\mathrm{QWP}\left(0^{\circ }\right)\mathrm{HWP}\left(\frac{b_{\theta }+\pi }{4}\right)|V\rangle ,\\ & & |B_{-}\rangle =\mathrm{QWP}\left(0^{\circ }\right)\mathrm{HWP}\left(\frac{b_{\theta }}{4}\right)|V\rangle . \end{array}$$

### 3.3. Quantum State Tomography

Quantum state tomography provides the benchmark technique for entanglement characterization, reconstructing unknown density matrices through informationally complete measurements. Experimental implementations employ either tetrahedral POVM or mutually unbiased base (MUB) schemes, performing 16 projective measurements at the single-copy level while recording coincidence counts. Statistical data processing combines maximum likelihood estimation (MLE) with constrained least squares (CLS), suppressing statistical noise and counting fluctuations while ensuring positive semidefinite, trace-normalized density matrices. The measurement set scale and total coincidence count jointly determine tomography accuracy. By quantifying error upper bounds via the Fisher information matrix spectral norm, measurement schemes can be pre-optimized to reduce fidelity uncertainty to ≤10^−3^, satisfying precision requirements for entanglement criteria and self-testing.

An *n*-qubit state characterization requires density matrix reconstruction:

(13) $$\begin{array}{c} \hat{\rho }=\frac{1}{2^{n}}\sum_{i_{1},i_{2},\ldots ,i_{n}=0}^{3}r_{i_{1},i_{2},\ldots ,i_{n}}{\hat{\sigma }}_{i_{1}}⊗{\hat{\sigma }}_{i_{2}}⊗\ldots ⊗{\hat{\sigma }}_{i_{n}} \end{array}$$

where the $4^{n}$ parameters $r_{i_{1},i_{2},\ldots ,i_{n}}$ are real numbers, with normalization $r_{0,0,\ldots ,0}=1$ determining $4^{n}-1$ independent parameters. The $\sigma_{i}$ (i=0,1,2,3) denote Pauli matrices, and ⊗ represents the tensor product between operators on individual qubit Hilbert spaces. The average coincidence count is $n_{v}=N\langle \psi_{v}|\hat{\rho }|\psi_{v}\rangle$. The tomographic reconstruction formula becomes

(14) $$\begin{array}{c} \hat{\rho }=\frac{\sum_{v=1}^{16}M_{v}n_{v}}{N}=\frac{\sum_{v=1}^{16}M_{v}n_{v}}{\sum_{v=1}^{4}n_{v}} \end{array}$$

Since density matrices must satisfy normalization ($\mathrm{Tr}(\hat{\rho })=1$) and Hermiticity (${\hat{\rho }}^{†}=\hat{\rho }$), direct application of Equation (14) may violate these physical constraints. We therefore employ numerical optimization to ensure that reconstructed matrices adhere to all physical requirements using maximum likelihood estimation [40].

### 3.4. Data Acquisition and Coincidence Counting

Data acquisition employed a coincidence counting unit recording photon coincidences at 1-s intervals per measurement basis. To suppress systematic errors from source intensity fluctuations and detector efficiency variations, all raw counts were normalized. Multiple measurement repetitions per basis reduced statistical fluctuations, with arithmetic means serving as valid coincidence counts.

## 4. Experimental Results

**Figure 3:**
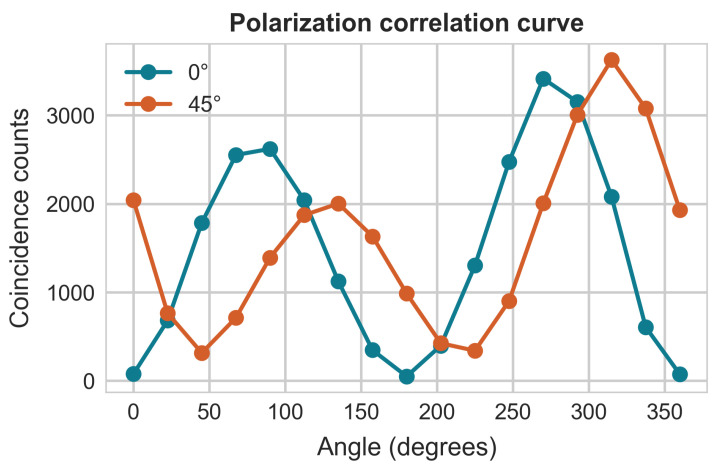
Polarization correlation curves at $0^{\circ }$ and $45^{\circ }$ measurement bases, where Angle represents Bob’s measurement angles corresponding to Alice’s measurement angles of $0^{\circ }$ and $45^{\circ }$, ranging from $0^{\circ }$ to $360^{\circ }$.

We implemented a self-testing protocol based on the sum-of-squares method to verify prepared two-qubit states. Polarization correlation measurements (Figure 3) demonstrated excellent agreement with theoretical predictions across different measurement bases. Repeated measurements for $\theta =30^{\circ }$, $45^{\circ }$, and $60^{\circ }$ states exhibited high stability and consistency, providing preliminary validation of state preparation reliability.

Experimental results confirm excellent agreement between prepared quantum states and theoretical predictions. Repeated measurements for different θ angles yielded highly stable data. Quantum state tomography reconstructions yielded fidelities exceeding 99% (Table 4), robustly validating our experimental approach.

These results demonstrate that our self-testing method offers significant advantages for certifying two-qubit states, providing an effective solution for verification requirements in quantum information processing and communication. Comprehensive error analysis confirms experimental reliability, supporting the practicality and precision of our approach.

**Figure 4:**
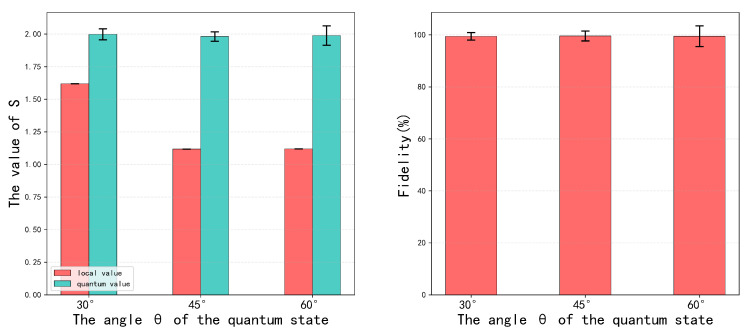
Experimental value of S. Left columns represent local bounds, while right columns represent nonlocal quantum values. The error bars are estimated according to the Poissonian counting statistics.

As shown in Figure 4, the classical values of S vary with parameter angle θ, while the quantum values of S for all angles exceed their classical counterparts. Entanglement concurrence measurements closely match theoretical predictions, indicating high-quality entanglement in prepared states. These results reinforce confidence in our experimental protocol and establish a solid foundation for further investigation of quantum state properties and applications.

**Figure 5:**
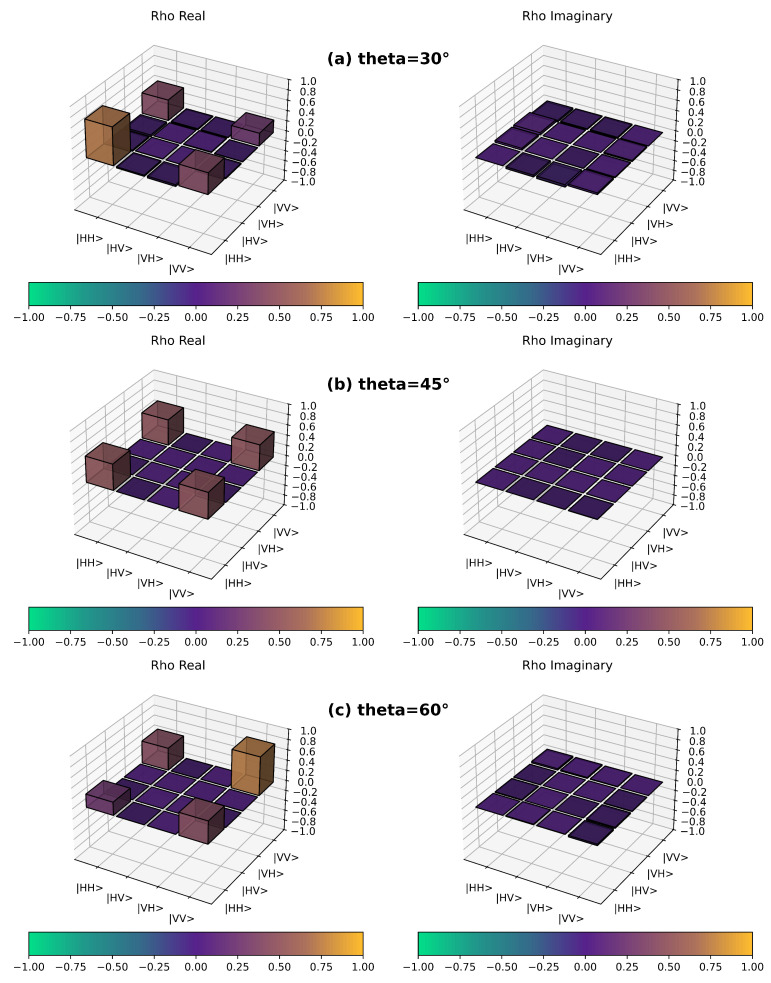
Quantum state tomography results for (**a**) $\theta =30^{\circ }$, (**b**) $\theta =45^{\circ }$, and (**c**) $\theta =60^{\circ }$. Left subpanels show real density matrix components, while right subpanels show imaginary components.

Quantum state tomography results for each parameter angle appear in Figure 5. The experimental data validate both the self-testing protocol’s effectiveness and the high-quality entanglement properties of prepared states. States generated via the sum-of-squares method demonstrate excellent performance in key metrics including Bell inequality violation, highlighting their practical value for quantum information applications.

## 5. Conclusions and Outlook

So far, the certification of network-generated states has been achieved with two independent entanglement sources [18], while multiphoton graph states, including GHZ and linear cluster states, have been self-tested using scalable stabilizer-based Bell inequalities [19,41]. Other studies focused on improving analytic self-testing bounds derived from Bell inequalities such as CHSH and Mermin, providing strong extractability guarantees for bipartite and tripartite states [42], or demonstrated the simultaneous certification of entangled states and projective measurements alongside randomness extraction [43]. In contrast to these efforts, we aimed to vertically enhance the core precision of device-independent certification. We achieved high-performance certification for a series of non-maximally entangled two-qubit states with fidelities exceeding 99.4%. Our experiment does not close the locality loophole and the measurement loophole that are encountered in most Bell-type experiments [44].

This work addresses the fundamental challenge of certifying non-maximally entangled states with high confidence within the device-independent framework by proposing and experimentally validating a complete solution. Theoretically, we construct optimal Bell inequalities for a given quantum state as a variational–Csum-of-squares optimization problem. Experimentally, using a room-temperature type-II PPKTP entanglement source and polarization-encoded photonic link, we implemented simultaneous quantum state tomography and optimal generalized Bell measurements on the same optical platform. For $\theta =30^{\circ }$, $45^{\circ }$, and $60^{\circ }$ states, we measured fidelities of 99.60%, 99.44%, and 99.47%, respectively, with the value of S significantly exceeding classical bounds. By focusing on vertically enhancing the fundamental capability of device-independent certification, we have achieved high-fidelity (>99.4%) self-testing for a family of non-maximally entangled two-qubit states and established performance benchmarks approaching theoretical limits. This advances device-independent quantum information processing by providing a universal methodological framework and experimental paradigm.

## Figures and Tables

**Table 1:** Conditional probability distribution p(a,b|x,y) for ideal CHSH self-testing.

(a,b)∖(x,y)	(0,0)	(0,1)	(1,0)	(1,1)
(0,0)	0.5	0.25	0.25	0.5
(0,1)	0.0	0.25	0.25	0.0
(1,0)	0.0	0.25	0.25	0.0
(1,1)	0.5	0.25	0.25	0.5

**Table 2:** Theoretical correlation expectation values for CHSH self-testing.

〈·, ·〉	*σ_Z_*	*σ_X_*
$\sigma_{Z}$	$1/\sqrt{2}$	$1/\sqrt{2}$
$\sigma_{X}$	$1/\sqrt{2}$	$-1/\sqrt{2}$

**Table 3:** Core optical components and their functions.

Component	Primary Function
Half-Wave Plate	Performs precise rotation of photon polarization to define the measurement basis.
Quarter-Wave Plate	Combined with the HWP to enable projections onto arbitrary elliptical polarization states.
Polarizing Beam Splitter	Routes photons to different paths based on their polarization (e.g., H or V), serving as the core device for polarization analysis.
Single-Photon Detector	Converts incoming single-photon signals into countable electrical pulses.
Coincidence Counter	Processes signals from the detectors to identify and record coincidence events from the same entangled photon pair via temporal correlation.

**Table 4:** Parameter angle vs. fidelity.

θ	Fidelity (%)
$30^{\circ }$	99.60 ± 0.084
$45^{\circ }$	99.44 ± 0.111
$60^{\circ }$	99.47 ± 0.229

## Data Availability

The original contributions presented in this study are included in the article. Further inquiries can be directed to the corresponding author.

## References

[1] Nielsen M.A., Chuang I.L. (2010). Quantum Computation and Quantum Information.

[2] Horodecki R., Horodecki P., Horodecki M., Horodecki K. (2009). Quantum entanglement. Rev. Mod. Phys..

[3] Lvovsky A.I., Raymer M.G. (2009). Continuous-variable optical quantum-state tomography. Rev. Mod. Phys..

[4] Flammia S.T., Gross D., Liu Y.-K., Eisert J. (2012). Quantum tomography via compressed sensing: Error bounds, sample complexity and efficient estimators. New J. Phys..

[5] Sangouard N., Simon C., Riedmatten H.D., Gisin N. (2011). Quantum repeaters based on atomic ensembles and linear optics. Rev. Mod. Phys..

[6] Amico L., Fazio R., Osterloh A., Vedral V. (2008). Entanglement in many-body systems. Rev. Mod. Phys..

[7] Blais A., Grimsmo A.L., Girvin S.M., Wallraff A. (2021). Circuit quantum electrodynamics. Rev. Mod. Phys..

[8] Kimble H.J. (2008). The quantum internet. Nature.

[9] Wehner S., Elkouss D., Hanson R. (2018). Quantum internet: A vision for the road ahead. Science.

[10] Eisert J., Friesdorf M., Gogolin C. (2015). Quantum many-body systems out of equilibrium. Nat. Phys..

[11] Mayers D., Yao A. (2003). Self testing quantum apparatus. arXiv.

[12] Supic I., Bowles J. (2020). Self-testing of quantum systems: A review. Quantum.

[13] Popescu S., Rohrlich D. (1992). Which states violate Bell’s inequality maximally?. Phys. Lett. A.

[14] Bancal J.-D., Navascués M., Scarani V., Vértesi T., Yang T.H. (2015). Physical characterization of quantum devices from nonlocal correlations. Phys. Rev. A.

[15] Acín A., Brunner N., Gisin N., Massar S., Pironio S., Scarani V. (2007). Device-independent security of quantum cryptography against collective attacks. Phys. Rev. Lett..

[16] Vidick T. (2014). Fully device-independent quantum key distribution. Phys. Rev. Lett..

[17] Colbeck R. (2009). Quantum and relativistic protocols for secure multi-party computation. arXiv.

[18] Agresti I., Polacchi B., Poderini D., Polino E., Suprano A., Supic I., Bowles J., Carvacho G., Cavalcanti D., Sciarrino F. (2021). Experimental robust self-testing of the state generated by a quantum network. PRX Quantum.

[19] Xu J.-M., Zhou Q., Yang Y.-X., Cheng Z.-M., Xu X.-Y., Ren Z.-C., Wang X.-L., Wang H.-T. (2021). Experimental self-testing for photonic graph states. Opt. Express.

[20] Bamps C., Pironio S. (2015). Sum-of-squares decompositions for a family of Clauser-Horne-Shimony-Holt-like inequalities and their application to self-testing. Phys. Rev. A.

[21] Einstein A., Podolsky B., Rosen N. (1935). Can quantum-mechanical description of physical reality be considered complete?. Phys. Rev..

[22] Bell J.S. (1964). On the Einstein Podolsky Rosen paradox. Physics.

[23] Aspect A., Dalibard J., Roger G. (1982). Experimental test of Bell’s inequalities using time-varying analyzers. Phys. Rev. Lett..

[24] Dong Q.-Q., Wang J.-Q. (2025). Deterministic high-dimensional quantum remote implementation via hybrid photonic encoding. Laser Phys..

[25] Qi J., Yu C.-Q., Yuan R.-Y., Yang Z., Ren B.-C. (2025). Entanglement-assisted logical Bell state measurement with linear optics. Opt. Laser Technol..

[26] Couteau C. (2025). Quantum computing using photons. Eur. Phys. J. A.

[27] Clauser J.F., Horne M.A., Shimony A., Holt R.A. (1969). Proposed experiment to test local hidden-variable theories. Phys. Rev. Lett..

[28] Sarkar S., Saha D., Kaniewski J., Augusiak R. (2021). Self-testing quantum systems of arbitrary local dimension with minimal number of measurements. npj Quantum Inf..

[29] Song D. (2024). Numerical simulation of Gedanken experiments involving violations of Bell’s inequalities. Int. J. Mod. Phys. C.

[30] Frérot I., Fadel M., Lewenstein M. (2023). Probing quantum correlations in many-body systems: A review of scalable methods. Rep. Prog. Phys..

[31] Abo S., Soubusta J., Jiráková K., Bartkiewicz K., Černoch A., Lemr K., Miranowicz A. (2023). Experimental hierarchy of two-qubit quantum correlations without state tomography. Sci. Rep..

[32] Hashem M., Mohamed A.-B.A., Haddadi S., Khedif Y., Pourkarimi M.R., Daoud M. (2022). Bell nonlocality, entanglement, and entropic uncertainty in a Heisenberg model under intrinsic decoherence: DM and KSEA interplay effects. Appl. Phys. B.

[33] Yang H., Zhao F., Fan X.-G., Ding Z.-Y., Wang D., Song X.-K., Yuan H., Zhang C.-J., Ye L. (2021). Estimating quantum steering and Bell nonlocality through quantum entanglement in two-photon systems. Opt. Express.

[34] Barizien V., Sekatski P., Bancal J.-D. (2024). Custom Bell inequalities from formal sums of squares. Quantum.

[35] Zhou Y.-C., Ma R.-L., Kong Z., Li A.-R., Zhang C., Zhang X., Liu Y., Jiang H.-T., Wu Z.-T., Wang G.-L. (2025). High-fidelity geometric quantum gates exceeding 99.9% in germanium quantum dots. Nat. Commun..

[36] Matsos V.G., Valahu C.H., Millican M.J., Navickas T., Kolesnikow X.C., Biercuk M.J., Tan T.R. (2024). Universal quantum gate set for Gottesman-Kitaev-Preskill logical qubits. arXiv.

[37] Liu W., Luo W., Tang Y., Wang G. (2025). Heralded and high-fidelity solid-state quantum Toffoli and Fredkin gates via practical microcavity-mediated photon scattering. Quantum Inf. Process..

[38] Chouraqui F. (2025). On the Realization of quantum gates coming from the Tracy-Singh product. Quantum Inf. Process..

[39] Qiu S., Xu Q., Zhou X. (2025). Detection of Weak Entanglement Based on Quantum Steering. Acta Phys. Sin..

[40] James D.F.V., Kwiat P.G., Munro W.J., White A.G. (2001). Measurement of qubits. Phys. Rev. A.

[41] Wu D., Zhao Q., Gu X.-M., Zhong H.-S., Zhou Y., Peng L.-C., Qin J., Luo Y.-H., Chen K., Li L. (2021). Robust self-testing of multiparticle entanglement. Phys. Rev. Lett..

[42] Zhang W.-H., Chen G., Peng X.-X., Ye X.-J., Yin P., Xiao Y., Hou Z.-B., Cheng Z.-D., Wu Y.-C., Xu J.-S. (2018). Experimentally robust self-testing for bipartite and tripartite entangled states. arXiv.

[43] Li X., Miao Y., Zhou W., Yu X., Song W., Wei Y., Gao F., Gao X., Gong Y.-X., Zhu S.-N. (2025). Experimental self-testing of complex projective measurements via elegant Bell inequality. Sci. China Phys. Mech. Astron..

[44] Aerts S., Kwiat P., Larsson J.-Å., Żukowski M. (1999). Two-photon Franson-type experiments and local realism. Phys. Rev. Lett..

